# Malrotated lateral radiographs do not allow for proper assessment of patellar height using the Caton‐Deschamps Index

**DOI:** 10.1002/jeo2.70663

**Published:** 2026-02-16

**Authors:** Moses Kamal Dieter El Kayali, Luis Vincent Bürck, Rosa Berndt, Alan Getgood, Clemens Gwinner, Lorenz Pichler

**Affiliations:** ^1^ Center for Musculoskeletal Surgery Charité – Universitätsmedizin Berlin Berlin Germany; ^2^ ASPETAR, Orthopaedic and Sports Medicine Hospital Doha Qatar; ^3^ Department of Orthopedics and Trauma‐Surgery Medical University of Vienna Vienna Austria

**Keywords:** Caton‐Deschamps Index, lateral knee radiograph, patellar height, patellar instability

## Abstract

**Purpose:**

To compare patellar height measurement (PHM) using the Caton‐Deschamps Index (CDI) on malrotated versus properly positioned lateral knee radiographs within the same patients. It was hypothesised that malrotation would significantly alter CDI values. A secondary aim was to establish an anterior–posterior distance (APD) cut‐off for malrotation corresponding to a minimally clinically important difference (MCID) of 0.1 in ΔCDI.

**Methods:**

This retrospective analysis included patients with lateral knee radiographs between January 2020 and March 2023 at a single academic institution. Patients were included if at least two radiographs were available: one with malrotation (APD ≥ 1 mm) and one properly aligned (APD < 1 mm). Radiographs with tilt, prior osseous surgery, or fractures were excluded. Patellar height was measured using the CDI. Inter and intrarater reliability were assessed via intraclass correlation coefficients (ICC). Paired *t*‐tests compared CDI values. Cases with a ΔCDI exceeding the MCID of ≥0.1 were identified. The APD cut‐off corresponding to a ΔCDI of 0.1 was determined by regression of ΔCDI on APD.

**Results:**

A total of 126 lateral knee radiographs from 63 patients (57% female) were analysed. Inter and intrarater reliability was excellent (ICC > 0.8 for all comparisons). The mean CDI was 0.96 ± 0.06 on properly aligned and 1.03 ± 0.08 on malrotated radiographs, with a significant mean difference of 0.07 ± 0.05 (*p* < 0.001). In 34.9% of patients, CDI differed by ≥0.1 between imaging conditions. Linear regression of ΔCDI on APD showed a slope of 0.016 per mm (*R*² = 0.57). The APD cut‐off corresponding to a ΔCDI of 0.1 was 6.3 mm (95% CI, 5.7–6.9 mm).

**Conclusions:**

Malrotation significantly alters PHM using the CDI on lateral knee radiographs. A clinically relevant difference of ≥0.1 in ΔCDI occurred in over one‐third of patients. Malrotated radiographs should be used with caution, and repeat imaging should be considered when CDI values approach clinical decision thresholds.

**Level of Evidence:**

Level III, diagnostic study.

**Abbreviations**
AI: artificial intelligence
CDI: Caton‐Deschamps Index
CI: confidence interval
ICC: intraclass correlation coefficient
MCID: minimal clinically important difference
PH: patellar height
PHM: patellar height measurement

## INTRODUCTION

Patellar height measurement (PHM) is critical for several musculoskeletal conditions, most notably patellofemoral instability [2, 4, 7, 18].

Numerous indices for PHM on lateral radiographs have been described [12, 22, 30, 43]. All such indices, however, depend on the quality of the lateral radiograph. Cadaveric and clinical studies have shown that rotational or tilting malposition of the knee can significantly alter apparent PHM, underscoring the need for a true lateral projection with superimposed posterior femoral condyles [20, 27].

In everyday orthopaedic practice, insufficient lateral radiographs with malrotation or tilt are common, and repeat imaging is not always performed [8]. Although it is recognised that malpositioning introduces error, no clearly defined threshold of malrotation currently exists beyond which PHM becomes clinically unreliable. As a result, clinicians lack practical guidance on when a lateral knee radiograph remains acceptable for PHM and when repeat imaging is warranted.

The primary aim of this study was therefore to compare, within the same patients, the Caton‐Deschamps Index (CDI) measured on properly aligned versus malrotated lateral knee radiographs. It was hypothesised that malrotation would produce statistically significant and clinically relevant differences in CDI [40]. The secondary aim was to establish an easy‐to‐measure cut‐off for malrotation, based on the anterior–posterior distance (APD) of the posterior femoral condyles [11], providing a clinically applicable threshold for determining when lateral knee radiographs are suitable for PHA or when repeat imaging should be performed.

## MATERIALS AND METHODS

This study was approved by the local institutional ethics committee (Nr. EA2/016/21) and conducted in accordance with the principles of the Declaration of Helsinki. Written informed consent was obtained from all participants before inclusion.

### Patients

In this retrospective analysis, a total of 502 patients who underwent knee surgery at our academic orthopaedic surgery centre between January 2020 and March 2023 were initially screened. Eligible patients had at least two lateral knee radiographs acquired during routine preoperative imaging: one demonstrating malrotation, defined as an APD of ≥1 mm and one with minimal or no malrotation, defined as an APD of <1 mm. Inclusion of both properly aligned and malrotated radiographs was possible because repeat imaging was frequently performed in clinical routine, either as quality‐control repeats during the same imaging session or as part of scheduled follow‐up examinations. If more than two eligible radiographs were available for the same patient, the pair with the shortest interval between the properly aligned and malrotated radiograph was selected to minimise potential temporal bias. Additional inclusion criteria were availability of written patient consent and complete patient records.

**Figure 1:**
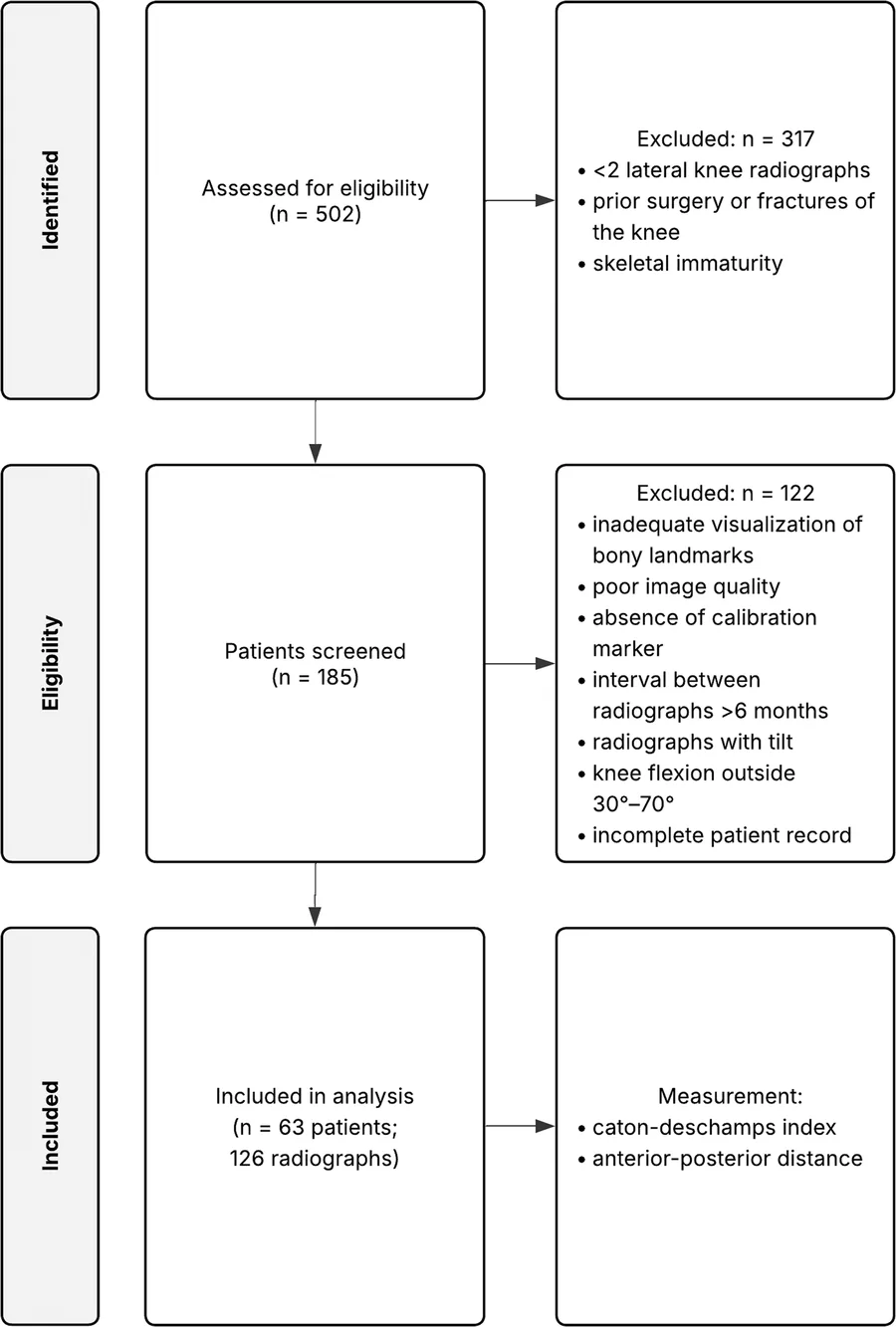
Flow chart of patient selection.

Patients were excluded based on the following criteria: inadequate visualisation of bony landmarks needed for measurement of patella height; excessive tilt, defined as adduction/abduction malpositioning with a proximal–distal distance >5 mm (distance between the most distal points of the medial and lateral femoral condyles); [19] poor image quality; absence of a calibration marker; more than 6 months between the two radiographs; history of prior bony surgery or fractures involving the knee; skeletal immaturity (open physis); knee flexion above 70° or below 30°; incomplete or missing patient records; or lack of written informed consent. For all included patients, clinical records were reviewed to confirm that no interventions or clinical events occurred between imaging sessions that could have influenced posture or arthrokinematics. The patient inclusion process is shown in Figure 1.

Demographic data collected included age at the time of surgery, date of radiograph, sex, side and body mass index, which were obtained from the patients′ electronic medical records.

### Radiographs

Lateral, weight‐bearing radiographs of the affected knee were obtained either during outpatient visits or at the time of surgical indication. Imaging followed a standardised acquisition protocol: patients were positioned standing with the knee flexed to approximately 45° [9], the detector oriented parallel to the sagittal plane, and the central x‐ray beam aimed at the patellofemoral joint line. All radiographs were calibrated using a reference marker of 25.4 mm (1 inch). Images were acquired on a digital radiography system (XGEO GC85A, Samsung).

### Measurement Technique

**Figure 2:**
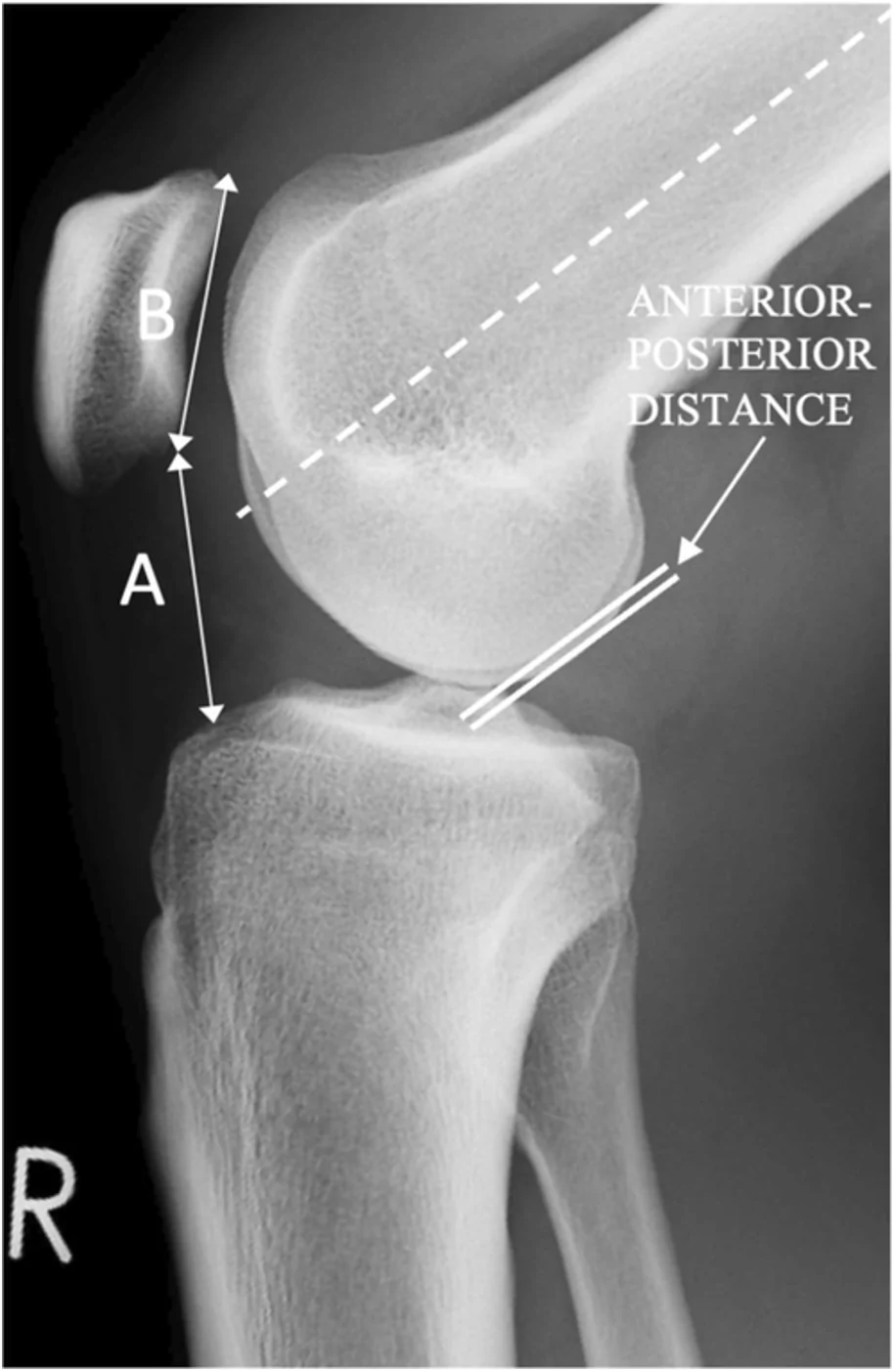
Measurement of CDI and APD. Lateral radiograph of a right knee showcasing measurements of patellar height using the CDI (A/B), and measurement of the APD. Radiograph is cropped for visualisation. APD, anterior–posterior distance; CDI, Caton‐Deschamps Index.

Patella height was determined on both the properly aligned and malrotated radiographs using the CDI [14]. This index compares the distance between the inferior edge of the patellar articular surface and the anterosuperior angle of the tibia on lateral radiographs, with the length of the articular surface of the patella (Figure 2). Although various techniques for PHM have been described [12, 14, 28, 30], the CDI was selected because it is simple to measure, is not affected by tibial tuberosity abnormalities, and is independent of the shape or length of the patella distal to the patellar articular surface [47]. Previous studies have demonstrated excellent inter and intraobserver reproducibility and strong agreement between radiographic and magnetic resonance imaging measurement [41, 42, 46], and the index is widely used in both clinical practice and research [1, 15, 17, 24, 52]. Moreover, it is the preferred method for PHM in clinical treatment guidelines for patellar instability [16, 29] and by the International Patellofemoral Study Group [37].

Malrotation was quantified using the APD between the posterior femoral condyles, as previously described [11]. APD has been validated as a reliable surrogate for rotation of the knee; in a sawbone model, Bixby et al. demonstrated that increasing APD correlates with increasing malrotation and significantly affects posterior tibial slope measurements [11], a finding subsequently confirmed in a clinical study [49]. To measure the APD, the femoral axis was defined by connecting the midpoints of two transverse lines drawn across the femoral shaft at separate levels, and the APD was measured perpendicular to this axis. Two additional lines were drawn parallel to this axis and placed as tangents to the most posterior aspects of the medial and lateral femoral condyles. The perpendicular distance between these lines was defined as the APD (Figure 2).

All measurements were performed independently by two observers who were blinded to each other′s results and to patient clinical data, using a picture archiving and communication system workstation (Centricity RIS‐I 4.2 Plus, GE Healthcare) and its integrated digital caliper tool, which allows distance measurements to the nearest 0.1 mm. APD measurements and subsequent view classification were completed before PHM to minimise circularity and recall bias. The order of image review was randomised across sessions. To assess both inter and intrarater reliability, each observer repeated all measurements after an interval of at least 14 days. For all subsequent analyses, the mean of both observers′ measurements was used.

### Statistics

All extracted data were compiled and summarised using Microsoft Excel (version 16.78, Microsoft Corporation). Statistical analysis was performed using IBM SPSS Statistics (version 28.0, IBM Corp.).

An a priori power analysis was performed using G*Power 3.1.9.6 for a two‐tailed paired‐samples *t*‐test. Based on a previous study reporting CDI values in the same patients, an effect size of Cohen′s *d* = 0.8 was assumed [20]. With *α* = 0.05 and a desired power of 0.95, the required sample size was *n* = 23 patient pairs. To ensure precise estimation of an APD cut‐off suitable for clinical application, a larger target sample of about 60 patients was chosen.

Descriptive statistics were reported as means ± standard deviations with 95% confidence intervals (CI). The Shapiro–Wilk test was used to assess normality, and Levene′s test was applied to evaluate the homogeneity of variances. For normally distributed variables, comparisons between properly aligned and malrotated radiographs were performed using a two‐tailed paired‐sample *t*‐tests. In cases of nonnormal distribution, the Wilcoxon signed‐rank test for related samples was used. Statistical significance was defined as *p* < 0.05 for all comparisons.

Inter and intrarater reliability was assessed by calculating the intraclass correlation coefficient (ICC), which was categorised as follows: slight (0–0.2), fair (0.21–0.4), moderate (0.41–0.6), good (0.61–0.8) or excellent (>0.8) [35].

The count and percentage of cases classified as outliers, defined as a paired difference in CDI between properly aligned and malrotated radiographs (ΔCDI) of more than 0.1 was reported [40]. A threshold of ≥ 0.1 was chosen because previous studies have identified this value as the minimal clinically important difference (MCID) for PHM [20, 27]. A linear regression analysis of ΔCDI on APD was performed to estimate the APD value corresponding to the MCID. Based on the regression‐derived APD threshold of 6 mm, the frequency of ΔCDI ≥ 0.1 was compared between patients with APD ≥ 6 mm and those with APD < 6 mm using a 2 × 2 contingency table. Fisher′s exact test, and the corresponding odds ratio were calculated to assess the statistical significance of this association. In addition, we recorded whether CDI values crossed the clinical patella alta threshold of 1.2 on the malrotated radiograph compared with the properly aligned radiograph [39].

## RESULTS

A total of 63 patients met the inclusion criteria (63 properly aligned lateral knee radiographs; 63 malrotated lateral knee radiographs) (Table 1). The mean interval between radiographs was 55 ± 25 days.

**Table 1:** Patients characteristics.

Parameter	Mean ± SD or no. (%)
Age, years	69.5 ± 10.2
BMI	29.3 ± 4.8
Sex	
Male	27 (43%)
Female	36 (57%)
Side	
Right	30 (48%)
Left	33 (52%)

Abbreviations: BMI, body mass index; SD, standard deviation.

Interrater reliability was excellent, with an ICC of 0.89 (95% CI, 0.83–0.93) for CDI and 0.93 (95% CI, 0.89–0.96) for APD. Intrarater reliability was similarly high, with ICCs of 0.91 (95% CI, 0.86–0.95) for Rater 1 and 0.94 (95% CI, 0.90–0.97) for Rater 2.

The mean CDI was 0.96 ± 0.06 (range, 0.83–1.17; CI, 0.94–0.97) in the properly aligned radiographs and 1.03 ± 0.08 (range, 0.85–1.31; CI, 1.01–1.05) in the malrotated radiographs, with a statistically significant mean difference of 0.07 ± 0.05 (CI, 0.05–0.09; *p* < 0.001).

**Figure 3:**
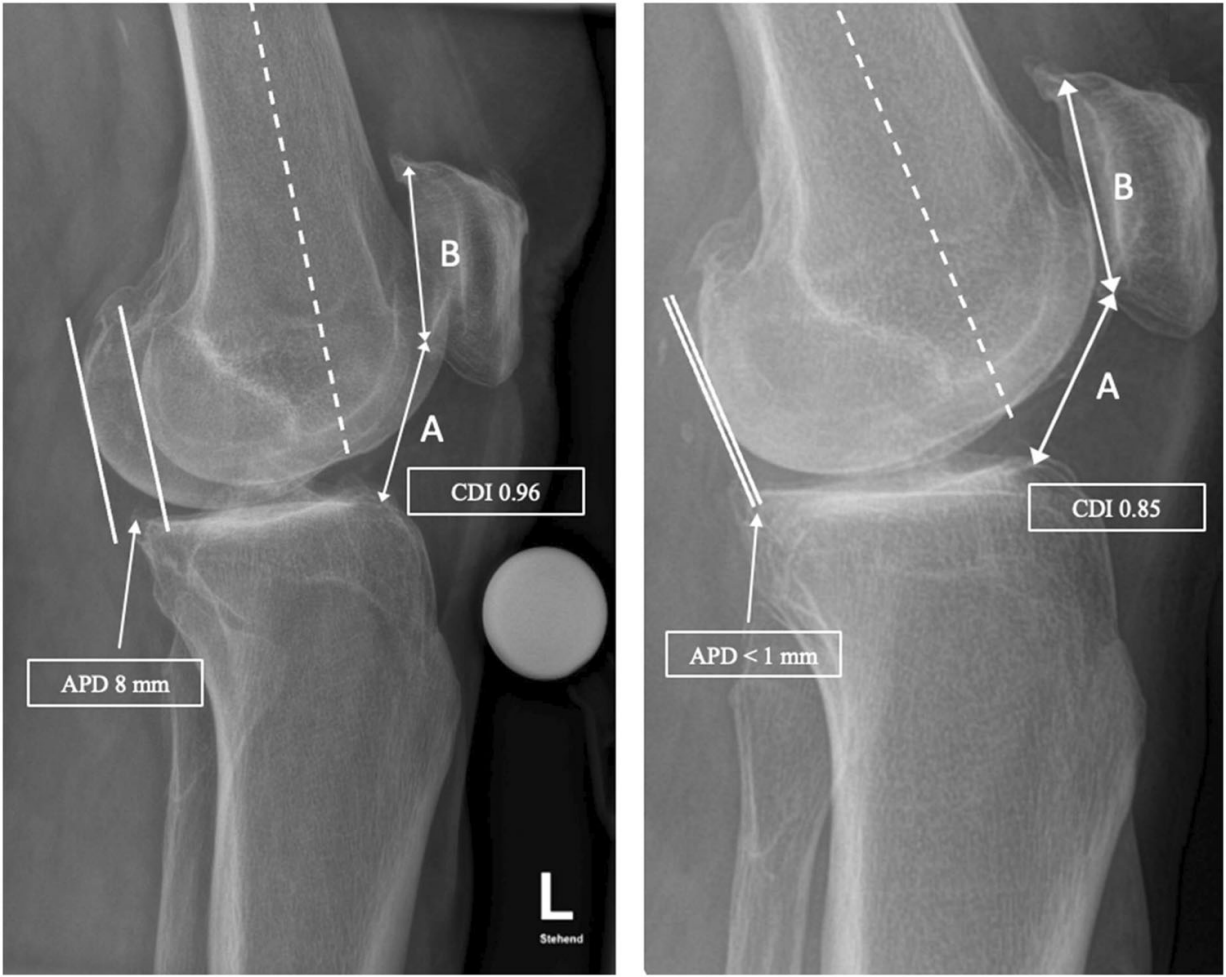
Effect of malrotation on CDI measurement in the same patient. Representative lateral knee radiographs from the same patient. Left: malrotated radiograph (APD = 8 mm). Right: properly aligned radiograph (APD < 1 mm). The measured CDI is >0.1 higher in the malrotated image, illustrating the systematic overestimation. Radiograph is cropped for visualisation. APD, anterior–posterior distance; CDI, Caton‐Deschamps Index.

The mean APD was 4.6 ± 2.6 mm (CI, 3.9–5.3 mm; range 1–11.8 mm) in the malrotated radiographs. A ΔCDI ≥ 0.1 was observed in 22 (34.9%) of patients. Figure 3 illustrates an example from the same patient, where malrotation resulted in a higher CDI.

**Figure 4:**
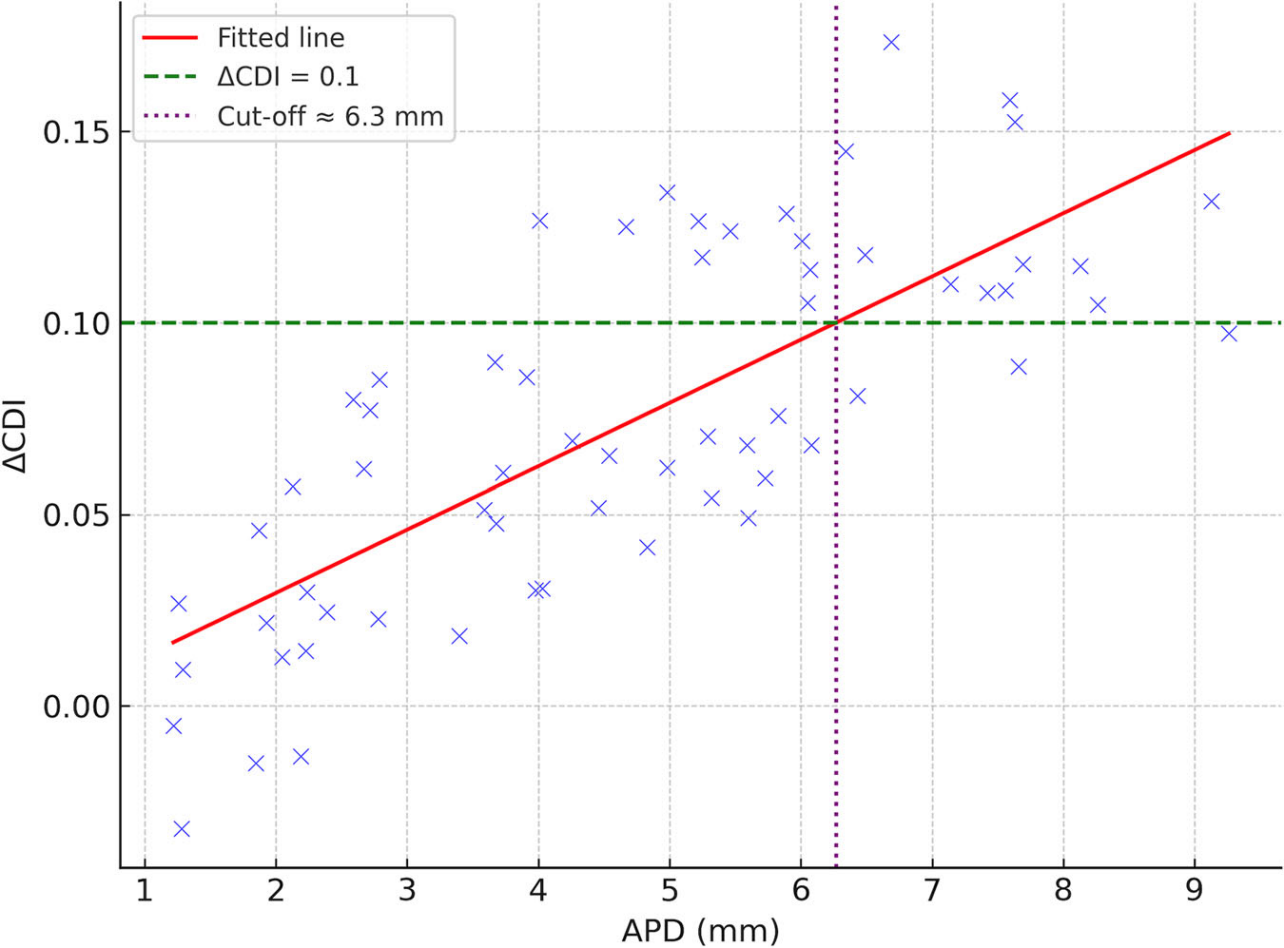
Linear regression of the association between ΔCDI and APD. Scatterplot of the change in ΔCDI between properly aligned and malrotated radiographs plotted against the APD on malrotated radiographs. Each dot represents one patient (*n* = 63). The red line shows the fitted linear regression (ΔCDI per mm of APD). The green dashed line marks the predefined MCID of ΔCDI = 0.1. The purple dotted line indicates the APD cut‐off (6.3 mm) at which ΔCDI reaches 0.1. APD, anterior–posterior distance; CDI, Caton‐Deschamps Index; MCID, minimal clinically important difference).

Linear regression of ΔCDI on APD showed a slope of 0.016 per mm (*R*² = 0.57). The APD cut‐off corresponding to a ΔCDI of 0.1 was 6.3 mm (CI, 5.7–6.9 mm) (Figure 4). A ΔCDI ≥ 0.1 occurred in 15 of 19 patients (78.9%) with an APD ≥ 6 mm, compared with 7 of 44 patients (15.9%) with an APD < 6 mm. Fisher′s exact test showed this difference to be statistically significant (*p* < 0.001), with an odds ratio of 19.82. Notably, in 5 patients (7.9%), the CDI exceeded the patella alta threshold of 1.2 only on the malrotated radiograph, while remaining below 1.2 on the properly aligned radiograph.

## DISCUSSION

The key finding of this study is that malrotation significantly influences PHM using the CDI on lateral knee radiographs. Within the same patients, malrotated radiographs yielded a mean CDI of 1.03 compared with 0.96 on properly aligned images, with a statistically significant mean difference of 0.07 (*p* < 0.001). More than one‐third of patients (34.9%) exhibited a change in CDI of ≥0.1, the MCID for PHM [27]. These findings confirm the study′s primary hypothesis that malrotation produces both statistically and clinically relevant differences in CDI measurements.

The secondary aim was to establish a simple, clinically usable threshold for malrotation. Using linear regression of ΔCDI on APD of the posterior femoral condyles, an APD cut‐off of 6.3 mm (95% CI, 5.7–6.9 mm) was identified as corresponding to a ΔCDI of 0.1. This threshold provides a practical criterion for deciding whether a lateral knee radiograph can be used for PHM or whether repeat imaging should be performed.

These results have direct clinical relevance, as accurate PHM is of importance in a range of musculoskeletal conditions [10, 25, 31, 33, 36, 48]. The association of patella height with patellofemoral instability is well documented [3, 4, 5, 6, 7, 13, 18, 21, 34]. Patella alta delays engagement of the patella within the trochlear groove during early flexion, increasing the unrestrained range of motion and facilitate lateral displacement and recurrent dislocation. It also increases the quadriceps moment arm, which elevates patellofemoral compression forces while simultaneously reducing contact area between 0° and 60° of flexion. Clinically, a CDI > 1.2 is commonly regarded as patella alta [39], and values above 1.2–1.3 have been proposed by various authors as an indication for tibial tubercle distalization, with a recommended postoperative target CDI of 1.0–1.1 [16, 29].

Therefore, inaccurate measurements due to malrotation could lead to misclassification of patellar height and potentially inappropriate surgical decision‐making. This observation is consistent with prior preclinical and clinical studies. In a cadaveric study using malrotated images, Huddleston et al. demonstrated that axial rotation produced statistically and clinically significant changes in CDI compared with true lateral radiographs [27]. Similarly, a clinical study comparing strictly lateral radiographs with those showing malrotation or tilt in 33 patients reported systematic overestimation of several patellar height indices, and warned that such deviations may predispose to diagnostic error and inappropriate surgical planning [20]. While both studies confirmed that malpositioning alters PHM, neither provided a practical parameter to quantify malrotation or a threshold at which measurements become unreliable.

The present study extends previous work by analysing paired radiographs within the same patients and, for the first time, identifying an objective and easy‐to‐measure APD cut‐off of 6 mm that clinicians can use to judge the adequacy of lateral radiographs before relying on CDI values for decision‐making. When this cut‐off is exceeded, images should not be used for PHM, and repeat imaging should be performed, as measurement errors are likely to surpass the MCID. The APD was first described by Bixby et al. in a sawbone model, where the distance between the posterior femoral condyles proved to be an excellent surrogate for quantifying rotational malalignment [11]. Although originally not introduced to study malpositioning effects on patellar height, measuring malrotation on lateral knee radiographs by the posterior femoral condylar distance has been adopted in several clinical studies as an objective metric of radiographic quality [23, 44, 49].

Inadequate radiographs are common in daily orthopaedic practice and are known to affect various key musculoskeletal parameters [23, 49]. In patients with patellofemoral instability, correctly rotated lateral radiographs are also essential for reliable assessment of trochlear dysplasia [32]. Reliance on such compromised measurements is not acceptable for clinical decision‐making. Looking ahead, artificial intelligence (AI) and machine learning offer promising tools to support clinicians in this regard. Automated quality‐control algorithms integrated into radiography systems could rapidly assess image alignment, flag radiographs exceeding defined quality thresholds and prompt repeat imaging when necessary. Studies have already demonstrated encouraging results for AI‐based radiograph classification and measurement, and despite current limitations, broader implementation could help ensure that only high‐quality images are used for musculoskeletal assessments [26, 38, 45, 50, 51].

## LIMITATIONS

This study has several limitations in addition to those inherent to its retrospective design. First, only a single patellar height index was evaluated. Although the CDI is the most widely used and recommended method for PHM [16, 29, 37], and clinical assessment often incorporates multiple indices in parallel, prior work has demonstrated that malpositioning can affect multiple indices [20], and the present findings are therefore likely applicable to other measurement techniques as well. Second, the analysis focused exclusively on rotational malalignment quantified by the APD. In clinical practice, lateral knee radiographs often exhibit a combination of malrotation and tilt (adduction/abduction malpositioning of the knee). Such combined malpositioning could produce even greater measurement errors and may necessitate stricter cut‐offs, which should be addressed in future studies. However, forming a separate subgroup for tilt analysis was not feasible in the present study, as the number of radiographs demonstrating isolated or combined tilt was too small to allow meaningful statistical evaluation. Third, although all included radiographs fell within a clinically acceptable knee flexion range, this range remains moderately wide and may introduce variability in PHM, as differences in knee flexion can lead to varying degrees of quadriceps activation that alter patellar position. In addition, centreing the x‐ray beam at the patellofemoral joint line rather than the posterior femoral condyles may introduce parallax effects due to beam divergence, which could influence measurements of the APD. Fourth, the study assesses the technical impact of radiographic malrotation on CDI measurements rather than clinical misclassification itself. Finally, the present cohort consisted predominantly of older patients undergoing imaging for degenerative knee conditions. In this population, osteophyte formation at the anterior tibial plateau may affect the identification of the tibial reference point used for CDI measurement and introduce variability that differs from malrotation‐induced error. However, such degenerative changes would be expected to affect both radiographs obtained in the same patient and, therefore, are unlikely to explain the systemic CDI differences observed in this study. Nevertheless, the exploration of the present findings to younger patients with patellofemoral instability should be made with caution.

## CONCLUSION

Malrotation significantly alters PHM using the CDI on lateral knee radiographs. A clinically relevant difference of ≥0.1 in CDI was observed in more than one‐third of patients. Malrotated lateral radiographs should be used with caution, and repeat imaging should be considered when CDI values approach clinical decision thresholds.

## AUTHOR CONTRIBUTIONS

Each named author has substantially contributed to conducting the underlying research and drafting this manuscript.

## CONFLICTS OF INTEREST STATEMENT

Alan Getgood: Consultant for Smith and Nephew, Stock ownership in Personalised Surgery and Kyniska Robotics.

## ETHICS STATEMENT

Please include the name of the institutional review board (IRB) and the approval number. If not applicable, please state so. The study protocol was approved by the local ethics committee (EA2/016/21), and the study was conducted in accordance with the Declaration of Helsinki. Written informed consent was obtained from all patients included in this study.

## Data Availability

The datasets generated and analysed during the current study are available from the corresponding author upon reasonable request.
